# Tools for analysis and conditional deletion of subsets of sensory neurons

**DOI:** 10.12688/wellcomeopenres.17090.1

**Published:** 2021-09-30

**Authors:** Sonia Santana-Varela, Yury D. Bogdanov, Samuel J. Gossage, Andrei L. Okorokov, Shengnan Li, Larissa de Clauser, Marta Alves-Simoes, Jane E. Sexton, Federico Iseppon, Ana P. Luiz, Jing Zhao, John N. Wood, James J. Cox

**Affiliations:** 1 Molecular Nociception Group, University College London, London, WC1E 6BT, UK; 2 Antibody and Vaccine Group, Centre for Cancer Immunology, MP127, University of Southampton Faculty of Medicine, Southampton, SO166YD, UK; 3 Institute for Biomedicine, Affiliated Institute of the University of Lubeck, Bolzano, Italy

**Keywords:** Advillin, dorsal root ganglia, primary sensory neuron, pain, somatosensation, Cre recombinase, diphtheria toxin

## Abstract

**Background: **Somatosensation depends on primary sensory neurons of the trigeminal and dorsal root ganglia (DRG). Transcriptional profiling of mouse DRG sensory neurons has defined at least 18 distinct neuronal cell types. Using an advillin promoter, we have generated a transgenic mouse line that only expresses diphtheria toxin A (DTA) in sensory neurons in the presence of Cre recombinase. This has allowed us to ablate specific neuronal subsets within the DRG using a range of established and novel Cre lines that encompass all sets of sensory neurons.

**Methods: **A floxed-tdTomato-stop-DTA bacterial artificial chromosome (BAC) transgenic reporter line (AdvDTA) under the control of the mouse advillin DRG promoter was generated. The line was first validated using a Na _v_1.8 ^Cre^ and then crossed to CGRP ^CreER^ (Calca), Th ^CreERT2^, Tmem45b ^Cre^, Tmem233 ^Cre^, Ntng1 ^Cre^ and TrkB ^CreER^ (Ntrk2) lines. Pain behavioural assays included Hargreaves’, hot plate, Randall-Selitto, cold plantar, partial sciatic nerve ligation and formalin tests.

**Results: **Motor activity, as assessed by the rotarod test, was normal for all lines tested. Noxious mechanosensation was significantly reduced when either Na _v_1.8 positive neurons or Tmem45b positive neurons were ablated whilst acute heat pain was unaffected. In contrast, noxious mechanosensation was normal following ablation of CGRP-positive neurons but acute heat pain thresholds were significantly elevated and a reduction in nocifensive responses was observed in the second phase of the formalin test. Ablation of TrkB-positive neurons led to significant deficits in mechanical hypersensitivity in the partial sciatic nerve ligation neuropathic pain model.

**Conclusions: **Ablation of specific DRG neuronal subsets using the AdvDTA line will be a useful resource for further functional characterization of somatosensory processing, neuro-immune interactions and chronic pain disorders.

## Introduction

Somatosensory neurons have been classified into distinct sets first on the basis of action potential velocity, later with histochemical markers, and more recently by single cell transcriptional profiling ^1– 4^. Gasser’s original observations of three sets of sensory neurons (A fibres, A delta fibres and C fibres) all of which could contribute nociceptive input, demonstrated the complexity of damage-sensing in the periphery. It was only in the 1960s that Perl demonstrated the existence of functionally specialised nociceptors ^5^. The debate about polymodality as opposed to modality-specific sensory neurons has been lively. It is clear that the *in vivo* properties of sensory neurons are altered when they are cultured, so that polymodality is often encountered in the dish and may be artefactual ^6^. The plasticity of sensory neurons caused by inflammatory mediators is also a complication.

The existence of defined transcripts that distinguish particular sets of sensory neurons provides an opportunity to analyse their function through genetic ablation studies. Recent studies suggest that the subdivisions of sensory neuron subsets identified in mice are in the main evolutionarily conserved in primates, which is of great significance for human studies ^3, 4, 7^. Thus analysis of rodent sensory neuron function is a useful tool for a better understanding of human somatosensation. Here we have used established and new Cre recombinase lines to ablate subsets of sensory neurons and carried out a preliminary analysis of the consequences for acute pain behaviour. It was important to rule out effects on other tissues, and so we developed a floxed stop diphtheria toxin mouse driven by a sensory neuron specific promoter, advillin. Advillin is selectively expressed in all sensory neurons and Merkel cells ^8^. This allowed us to ablate only sensory neurons with no effect on other tissue that may express the same transcripts as sensory neurons but in which advillin and hence diphtheria toxin is not expressed.

## Methods

### Ethical considerations

All experiments were performed in accordance with the UK Animals (Scientific Procedures) Act 1986 with prior approval under a Home Office project licence (PPL 70/7382). This study is reported in line with the ARRIVE guidelines ^9^.

### Mouse husbandry

The study took place at University College London in 2018–2021. Mice were kept on a 12-hr light/dark cycle with cage enrichment (e.g. disposable igloos) and provided with food and water *ad libitum*. Surgical procedures were performed by trained researchers and under aseptic conditions. For genotyping, genomic DNA was isolated from ear tissue for polymerase chain reaction (PCR) using GoTaq DNA Polymerase (Promega) (see *Extended data*, Supplementary Methods for primer sequences and cycling conditions ^9^). Cre ^ER/ERT2^ mouse lines received 200 μl doses i.p. of a 1% tamoxifen solution on five consecutive days (once per day) between 8–10 weeks of age. Tamoxifen was prepared in 15% ethanol/85% sunflower oil.

### Mouse lines

****Advillin* Flox-tdTomato-Stop-DTA BAC transgenic (AdvDTA).*** We generated a floxed-tdTomato-stop-DTA bacterial artificial chromosome (BAC) transgenic reporter line under the control of the mouse *Advillin* ( *Avil*) promoter. The construct was generated using a recombineering protocol described in detail by Copeland *et al*. ^10^ ( *Extended data,* Supplementary Figure 1 ^9^). BAC clone RPCI23-424F19 containing part of the *Advillin* gene was used as a template to prepare a targeting construct. To construct the shuttle vector for recombineering the 5'-homology arm (432 bp) and the 3'-homology arm (274 bp) flanking exon 2 of the *Advillin* gene were cloned into a vector containing a floxTomatoDTA cassette and a Kanamycin cassette, the latter being flanked by flippase recognition target (FRT) sites. The floxTomatoDTA cassette consists of the sequence encoding tdTomato protein followed by three SV40 polyadenylation signals and flanked by the loxP sites. This sequence was fused with the sequence encoding DTA protein followed by bGH and SV40 polyA signals. The start codon in the exon 2 of the *Advillin* gene corresponds to the start codon of tdTomato. The completed shuttle construct was sequenced and the targeting cassette was isolated from the plasmid by AscI/PacI digest.

EL250 E. coli cells transformed with the BAC clone were co-transformed with the shuttle targeting construct and recombination was induced by heat-shock according to the recombineering protocol. The induced bacterial clones were isolated by growing on Kanamycin-containing plates. The Kanamycin resistance cassette was removed by inducing Flp-recombinase expression by arabinose. The final construct was verified by PCR and analytical restriction digests. The resulting BAC containing the targeting cassette was sent to Cyagen for pronucleus injection. The founder line was bred with wild-type C57BL6/J mice to obtain germline transmission.

### Cre and floxed stop tdTomato reporter lines

The following lines were provided as generous gifts: CGRP ^CreER 11^, TrkB ^CreER^ ^12^ (Jackson Lab stock number 027214), Th ^CreERT2^ ^13^ (Jackson Lab stock number 025614), Ntng1 ^Cre^ ^14^ and Rosa-CAG-flox-stop-tdTomato ^15^ (Jackson Lab stock number 007905). The Na _v_1.8 ^Cre^ line was in house and previously described ^16^.

Tmem45b ^Cre^ and Tmem233 ^Cre^ lines were generated by Cyagen on a C57BL/6 background. The Tmem45b ^Cre^ line is a BAC transgenic in which a fragment containing the Cre-SV40 pA together with Frt-Kanamycin cassette-Frt was introduced in exon 2 of the murine *Tmem45b* gene within BAC clone RP24-249I6. The Kanamycin cassette was removed from the recombinant BAC by Flp-mediated recombination. The recombinant clone was verified by PCR and end-sequencing and then injected into fertilized eggs for transgenic mouse production. Pups were genotyped by PCR using GoTaq DNA Polymerase (Promega) (see *Extended data*, Supplementary Methods for primer sequences and cycling conditions ^9^). Founder ‘R’ was used for further breeding and characterization.

The Tmem233 ^Cre^ line is a constitutive knockin line in which the Kozak-Cre-pA cassette replaced the ATG start codon of the murine *Tmem233* gene. The Cre is under the control of *Tmem233* regulatory elements with homozygotes being knockouts of the *Tmem233* gene. The homology arms of the targeting vector were generated by PCR using BAC clones RP23-182O14 and RP23-375I17. In the targeting vector the Neo cassette was flanked by Rox sites and DTA was used for negative selection. The final constitutive KI allele was obtained after Dre-mediated recombination with C57BL/6 ES cells used for gene targeting. Pups were genotyped by PCR using GoTaq DNA Polymerase (Promega) (see *Extended data*, Supplementary Methods for primer sequences and cycling conditions ^9^). 

### Behavioural tests

For all behavioural assays, the animals in the test groups carried both the Cre gene and the AdvDTA BAC. Animals in the control groups were negative either for the Cre gene or the AdvDTA BAC (see each figure legend for specific details). Sample sizes (1 unit=1 animal) were calculated based on our previous experience for each assay ^17^ and animals were excluded from the study if they became unwell. All data points were included in the statistical analyses. Animals were acclimatized to the behavioural equipment for at least 2 days prior to testing; control and test groups were assayed on the same day. Observers who performed behavioural experiments were blind to the genotype of the animals. Animals were selected and placed into the apparatus randomly by an independent experimenter. The unblinding of each group followed input of the raw behavioural data into the analysis software. Experiments were conducted using both male and female adult transgenic mice and wild-type littermates (all >7 weeks in age).

Mechanical nociceptive thresholds were measured using a modified version of the Randall Selitto test for mice (Ugo Basile) that applies pressure to the tail with a 500g cut-off ^17, 18^. Punctate mechanical sensitivity was measured using the up-down method for obtaining the 50% threshold using von Frey hairs (Ugo Basile stand; Aesthesio hairs), as previously described ^17, 19^. Thermal nociceptive thresholds were determined by measuring paw-withdrawal latency using the Hargreaves’ apparatus (IITC Life Science) ^17, 20^ with a ramp of 1.5°C/s and a 30-s cut-off and also by use of the 50°C hot-plate test (Ugo Basile) ^21^. The response to noxious cold was measured using the cold plantar assay (Ugo Basile) ^22^. The rotarod test (IITC Life Science) was performed as described in 23. The formalin model was performed by intraplantar injection (subcutaneously) of 20 μl of a 5% formalin solution into the left hindpaw after a 15 min acclimatization to individual observation cages. Time spent engaged in nociceptive behaviour (biting or licking the affected region) was recorded over 1 hr immediately after injection. The video recordings were sampled for 10 sec at 1 min intervals using a Python script and scored for the presence or absence of nocifensive behaviours toward the injected hind paw (licking, biting, flinching). Data is expressed as a percentage of nocifensive responses out of all responses. The early acute phase (Phase 1, 0-10 min) and the latter inflammatory phase (Phase 2, 10–60 min) were separately analysed.

### Partial sciatic nerve ligation model

Surgical procedures were performed under isoflurane anaesthesia (inhalation) (2–3%). A partial nerve injury in adult mice was induced by tying a tight ligature with 6-0 silk suture around approximately 1/3 to 1/2 the diameter of the sciatic nerve, similar to the approach described in rats ^24^.

### *In situ* hybridization (ISH) sample preparation and RNAscope

Animals were deeply anesthetized with pentobarbital (i.p.) and transcardially perfused with heparinized saline (0.9% NaCl) followed by 25 ml of cold 4% paraformaldehyde in phosphate-buffered saline (pH 7.4). Dorsal root ganglia were extracted from the lumbar area and post-fixed with the same fixative solution for 2 hours at 4 °C before being embedded in cryopreservative solution (30% sucrose) overnight at 4°C. Tissue samples were then placed in optimal cutting temperature (OCT) compound blocks for posterior sectioning by cryostat. 11 μm thick sections were mounted onto Superfrost Plus (Fisher Scientific) slides, allowed to freeze-dry overnight at -80 °C, for an immediate use, or were stored at −80 °C for no longer than a month for subsequent experiments.

*In situ* hybridization was performed using the RNAscope system (Advanced Cell Diagnostics) following a two-day protocol for fresh-frozen samples with 1hr post-fixing with 4% paraformaldehyde (PFA) in phosphate buffered saline (PBS) at 4°C and stepwise dehydration with 50%, 70% and 100% ethanol. Tissue pre-treatment consisted of hydrogen peroxide and protease IV (10 and 20 min, respectively) at room temperature (RT). Following pre-treatment, probe hybridization and detection with the Multiplex Fluorescence Kit v2 were performed according to the manufacturer’s protocol.

Probes included Tmem233 (#519851), Tmem45b (#420461-c3), NEFH (#443671 or #443671-c4), Th (#317621-c4), CGRP (Calca #417961-c3), Ntng1 (#488871-C2), Ntrk2 (#423611-c3) and tdTomato (#317041-c4). Ribonucleic acid (RNA) localisation was detected with Alexa Fluor™ 488 Tyramide (Cat# B40953, green, for all target gene RNA products) and Opal 570 (Cat# FP1488001KT, red, when a probe against tdTomato was used) fluorochrome dyes (Perkin Elmer) compared to DAPI staining (nuclei) or TS-coumarin (TS405, blue, Cat# NEL703001KT, Perkin Elmer) used for neurofilament heavy chain (NEFH). *In situ* hybridisation slides were mounted using Prolong Gold (ThermoFisher Scientific #P36930).

Fluorescence was detected using Zeiss LSM 880 Airyscan microscope. Images were taken at 10x or 20x magnification with 4x averaging, airyscan processed, stitched whenever required and exported as 16-bit uncompressed tiff files for further basic editing in Adobe Lightroom v6 (Adobe) on colour calibrated iMac retina monitor. Final images were exported as jpeg files with 7,200 pix on longest side at 300 ppi.

### RNA extraction and real-time qPCR

RNA was extracted from dorsal root ganglia tissue using TRIzol Reagent (Life Technologies) and Purelink RNA micro kit (Thermo Fisher) and then reverse transcribed according to standard protocols. Real-time RT-PCR using technical triplicates was performed with the BioRad CXF Connect ^TM^. PCR was carried out using the Universal SYBR Green Supermix protocol (Bio-Rad) and primers to *Scn10a* and *Gapdh* (see *Extended data*, Supplementary Methods for primer sequences and cycling conditions ^9^). *Scn10a* expression was compared with that of *Gapdh* measured on the same sample in parallel on the same plate, giving a CT difference (ΔCT) for *Gapdh* minus *Scn10a*. Mean and standard error were performed on the ΔCT data and converted to relative expression levels (2ˆΔCT).

### Statistics

Data was analysed using GraphPad Prism 9 (GraphPad Software, Inc), and results presented as mean ± SEM with n referring to the number of samples tested per group, as indicated in figure legends. SOFA Statistics is an alternative open access package that can be used.

## Results

**Figure 1.**
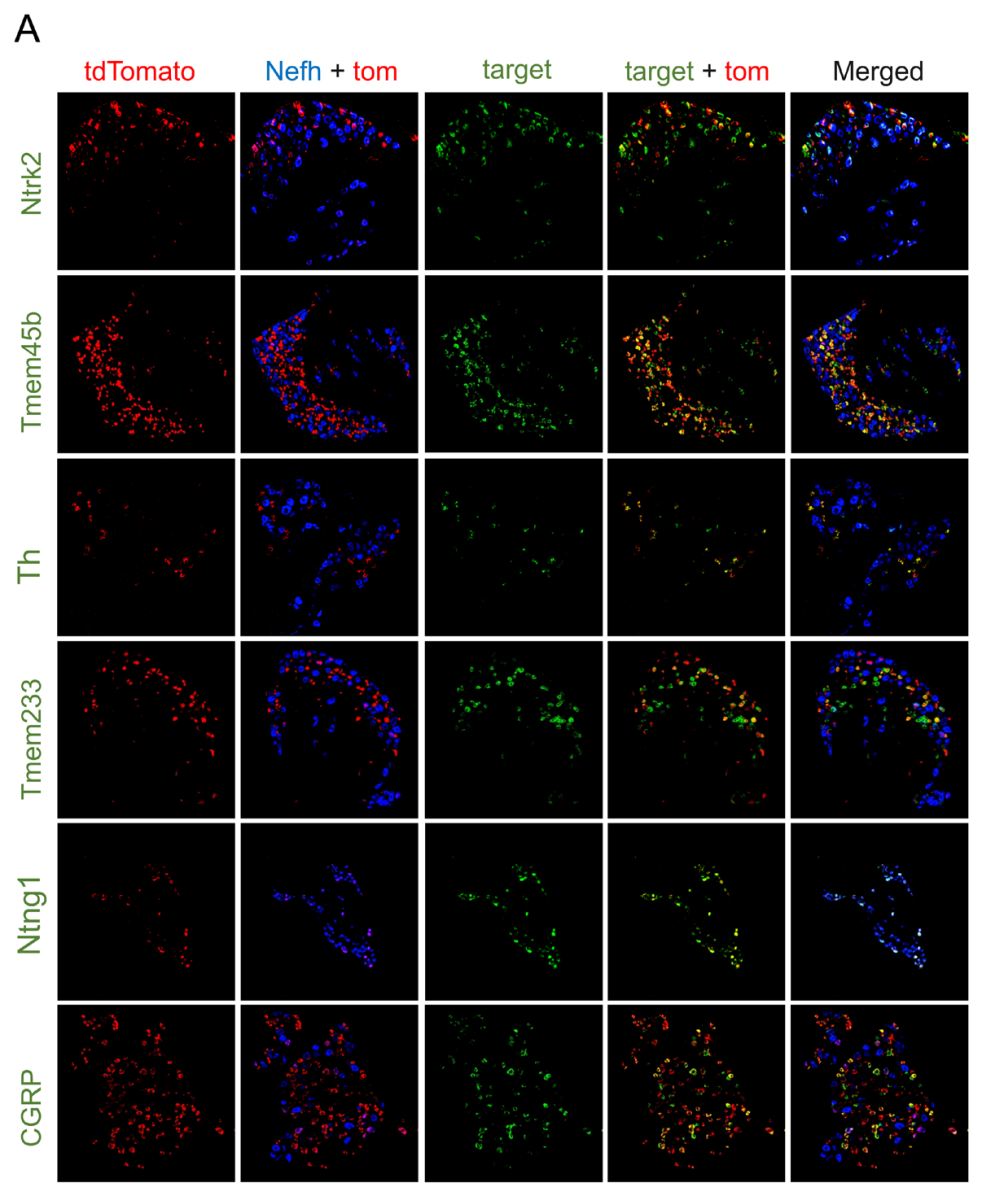
RNAscope analysis of Cre activity in dorsal root ganglia (DRG) neurons. Expression of tdTomato (red), Nefh RNA (detected with TS405, blue) and target marker gene RNA (detected with AF488, green). Cre lines indicated on the left: TrkB ^CreER^ (Ntrk2), Tmem45b ^Cre^, Th ^CreERT2^, Tmem233 ^Cre^, Ntng1 ^Cre^, CGRP ^CreER^ (Calca). These lines were crossed to a Rosa-CAG-flox-stop-tdTomato line and lumbar DRGs isolated from adult mice for RNAscope analyses.

Single cell RNA sequencing of mouse dorsal root ganglia neurons has provided an unbiased classification of neuronal subpopulations based on their genetic profiles ^3, 4^. Using these data, we selected marker genes that could be used to drive expression of Cre recombinase in restricted DRG neuron populations to enable further functional analyses of neuronal subsets ( Table 1). We recruited existing lines and generated new lines for further analyses (see Materials and Methods): Cgrp ^CreER^ (Calca), Th ^CreERT2^, Scn10a ^Cre^ (Na _v_1.8), Tmem45b ^Cre^, Tmem233 ^Cre^, Ntng1 ^Cre^, and TrkB ^CreER^ (Ntrk2). Lines were crossed to a Rosa-CAG-flox-stop-tdTomato line and following tamoxifen administration for the CreER/CreERT2 lines, lumbar DRGs were isolated from adult mice for RNAscope testing. Figure 1 shows representative images of tdTomato expression (i.e. following Cre activity) and expression of the ‘target gene’ (i.e. an *in situ* probe against each marker gene mRNA) ^9^. For each line tested, there was a large overlap between tdTomato expression and the target, indicating that Cre activity was in general consistent with expression of the endogenous marker gene. Figure 1 also shows expression of neurofilament ( *Nefh*) mRNA, which is expressed in NF1, NF2, NF3, NF4, PEP1.2 and PEP2 neurons, according to single cell RNAseq data ^3, 4, 25^. Comparison of Cre activity (tdTomato expression) and *Nefh* expression was as predicted from single cell RNAseq data ( Table 1 and Figure 1). For example, Tmem45b Cre activity was present in Nefh-negative neurons; Ntng1 Cre was active in Nefh-positive neurons; and Calca Cre activity was in both Nefh-positive and -negative neurons.

**Table 1.** Expression profile of selected marker genes within mouse dorsal root ganglia (DRG) neurons.

	PSPEP6 TrpM8.1	PSPEP7 TrpM8.2	PSPEP8 TRPM8.3	PSPEP5 PEP1.1	PSPEP2 PEP1.2	PSPEP4 PEP1.3	PSPEP3 PEP1.4	PSPEP1 PEP2	PSNF1 NF1	PSNF2 NF2	PSNF2 NF3	PSNF3 NF4	PSNP1 TH	PSNP3 NP1.1	PSNP2 NP1.2	PSNP4 NP2.1	PSNP5 NP2.2	PSNP6 NP3
*Calca*																		
*Th*																		
*Pvalb*																		
*Scn10a*																		
*Tmem45b*																		
*Tmem233*																		
*Ntng1*																		
*Ntrk2*																		
*Avil*																		

The PSPEP, PSNF and PSNP nomenclature of neuron types on top refers to the classification presented in Zeisel *et al*., 2018 (available at mousebrain.org) ^4^. The TrpM8, PEP, NF, TH and NP nomenclature represents the relationship of these neuron types to the classification presented in Usoskin *et al*., 2015 ^3^. PEP (peptidergic); NF (neurofilament); NP (non-peptidergic); TH (tyrosine hydroxylase). Marker genes listed in far center column. Table adapted from 25.

### *Advillin* Flox-tdTomato-Stop-DTA BAC transgenic

The library of validated Cre lines enables specific DRG neuronal populations to be ablated via Cre-dependent expression of Diphtheria toxin A (DTA). However, as many of the Cre lines drive expression in tissues outside of the DRG, we generated a floxed-tdTomato-stop-DTA BAC transgenic reporter line under the control of DRG-enriched mouse *Advillin* ( *Avil*) promoter to enable restricted ablation ^26^ ( *Extended data,* Supplementary Figure 1 ^9^). The targeting construct used to generate the line consists of a tdTomato fluorescent reporter gene and transcriptional stop sequences that are flanked by loxP sites with a DTA sequence downstream. Advillin is expressed in all sensory neurons and has minimal expression elsewhere in the nervous system ^27^. In Cre negative animals, the tdTomato gene is expressed with transcription of the DTA prevented by the upstream transcriptional stop sequences. In Cre positive animals, the tdTomato and transcriptional stop sequences are excised and the DTA is transcribed leading to toxin production and cell ablation.

**Figure 2.**
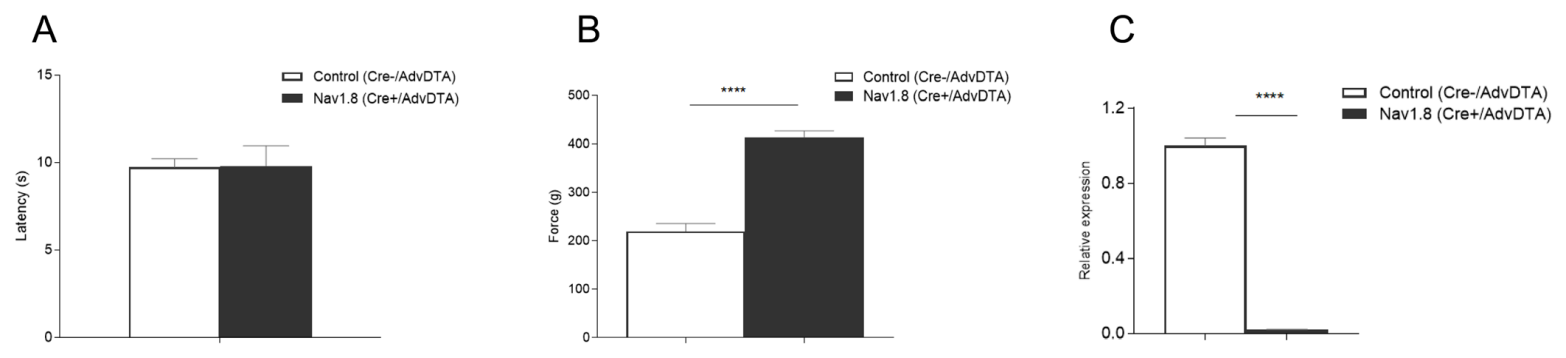
Validation of the *Advillin* Flox-tdTomato-Stop-DTA (AdvDTA) BAC transgenic. ( **A**) Na _v_1.8 Cre+/AdvDTA mice show normal responses to noxious thermal stimuli in the Hargreaves’ test (t-test: p>0.05 (ns)); Na _v_1.8 Cre-/AdvDTA n=6, Na _v_1.8 Cre+/AdvDTA n=5;. ( **B**) Na _v_1.8 Cre+/AdvDTA mice show significantly impaired noxious mechanosensation in the Randall-Selitto test (t test: p<0.0001); Na _v_1.8 Cre-/AdvDTA n=6, Na _v_1.8 Cre+/AdvDTA n=5. ( **C**) qPCR data shows a 97.5% decrease in levels of Na _v_1.8 mRNA following ablation of the Na _v_1.8-positive subset of sensory neurons in Na _v_1.8 Cre+/AdvDTA mice (t test: p<0.0001). Na _v_1.8 Cre-/AdvDTA n=3, Na _v_1.8 Cre+/AdvDTA n=3.

To validate this new reporter we crossed it with the Na _v_1.8 ^Cre^ line and were able to recapitulate the behavioural phenotype observed in our earlier work in which the Na _v_1.8 ^Cre^ was bred to a Rosa floxed stop DTA knockin line ^28^. We used the Hargreaves’ test to apply a latent heat stimulus to the hind paw and recorded the latency to a nocifensive response. We also used the Randall-Selitto test to apply steadily increasing mechanical pressure to the tail until a withdrawal response was observed. We showed that mice lacking the Na _v_1.8 positive population of sensory neurons have unimpaired responses to noxious heat ( Figure 2A), but responses to noxious mechanical stimuli are substantially impaired ( Figure 2B) suggesting that we successfully ablated Na _v_1.8 containing neurons in the BAC transgenic line. Real-time qPCR in DRG isolated from the Cre+/AdvDTA+ mice confirmed the ablation with a 97.5% decrease in Na _v_1.8 mRNA levels compared to WT DRGs ( Figure 2C).

### Pain behaviour tests following restricted ablation of subpopulations of DRG neurons

**Figure 3.**
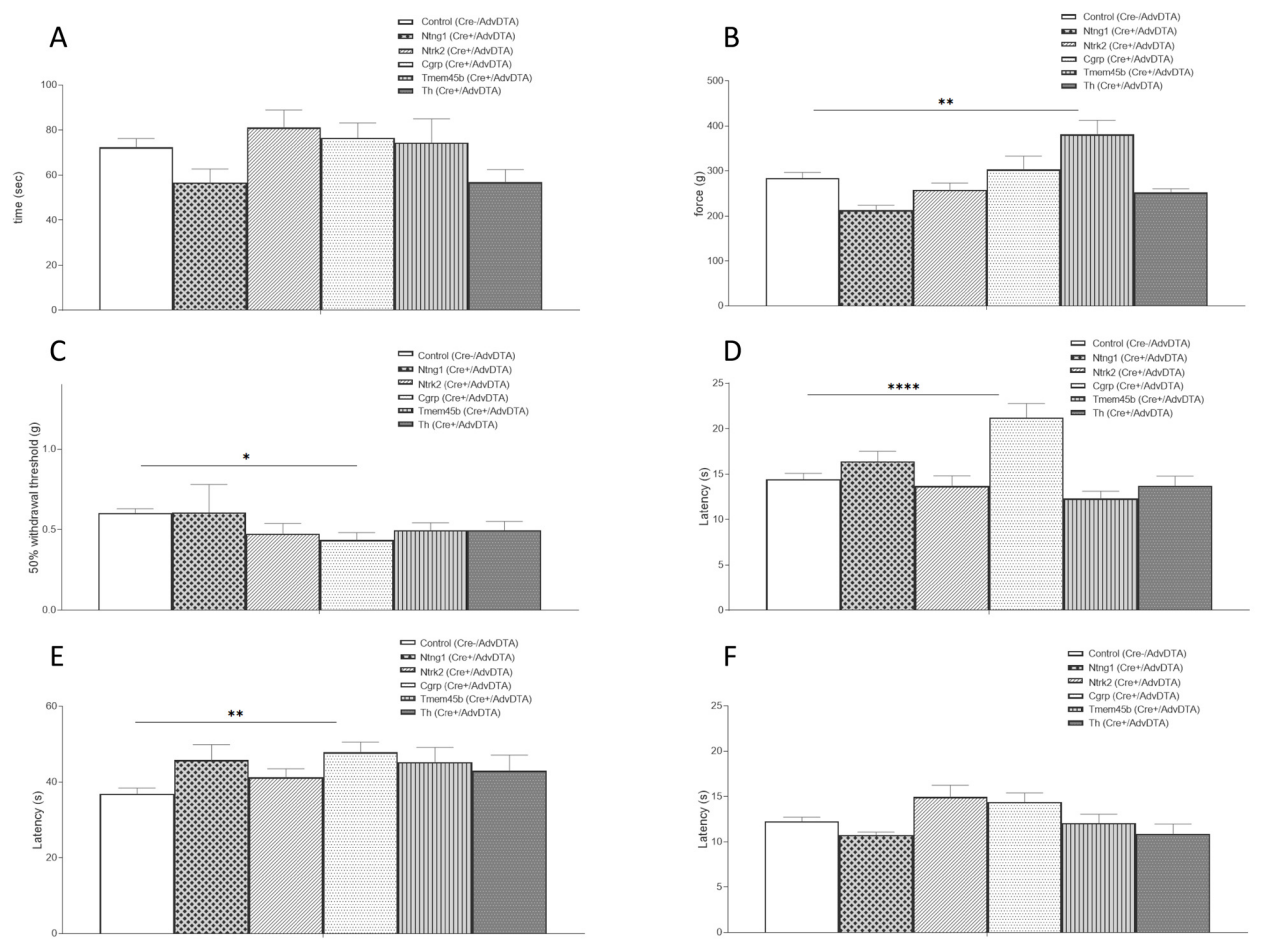
Behavioural analyses following ablation of subpopulations of dorsal root ganglia neurons. ( **A**) All AdvDTA+/Cre+ mouse lines tested show normal motor co-ordination as assessed by the rotarod test (ANOVA, p>0.05 (ns)); ( **B**) AdvDTA+/Tmem45b Cre+ mice show significantly impaired noxious mechanosensation in the Randall-Selitto test (ANOVA, p<0.005); ( **C**) Increased sensitivity to punctate mechanical sensitivity was observed in the AdvDTA+/CGRP Cre+ line (ANOVA, p<0.05); ( **D**) AdvDTA+/CGRP+ mice were hyposensitive to noxious heat in the Hargreaves’s test (ANOVA, p<0.0001) and the ( **E**) hot plate test (ANOVA, p<0.005); ( **F**) All AdvDTA+/Cre+ mouse lines tested show normal responses in the cold plantar assay (ANOVA, p>0.05 (ns)). AdvDTA+/Cre- (n=52-54), AdvDTA+/Ntng1 Cre+ (n=5), AdvDTA+/Ntrk2 Cre+ (n=14), AdvDTA+/CGRP Cre+ (n=15-16), AdvDTA+/Tmem45b Cre+ (n=14) and AdvDTA+/Th Cre+ (n=7-8).

**Figure 4.**
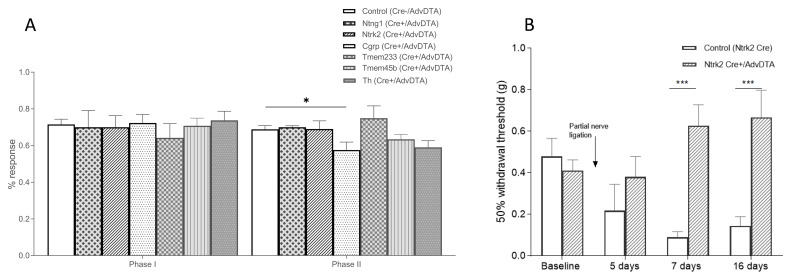
Inflammatory and neuropathic pain behaviour following ablation of subpopulations of DRG neurons. ( **A**) Formalin test in all AdvDTA+/Cre+ mouse lines tested show normal phase 1 nocifensive responses to intraplantar injection of formalin. Ablation of CGRP+ neurons results in reduced responses in the second phase of the formalin test (ANOVA, p<0.05). AdvDTA+/Cre- (n=43), AdvDTA+/Ntng1 Cre+ (n=4), AdvDTA+/Ntrk2 Cre+ (n=6), AdvDTA+/CGRP Cre+ (n=13), AdvDTA+/Tmem233 Cre+ (n=9), AdvDTA+/Tmem45b Cre+ (n=13) and AdvDTA+/Th Cre+ (n=8). ( **B**) Mechanical sensitivity following the partial sciatic nerve ligation model in Ntrk2 Cre (n=9) and AdvDTA+/Ntrk2 Cre+ (n=10) mice (ANOVA, p<0.0005).

Following validation of the AdvDTA reporter we crossed this BAC transgenic line to five additional Cre-expressing lines with distinct expression profiles across DRG neuronal subsets ( Table 1). As expected, motor co-ordination as assessed by the rotarod test, was normal for all lines compared to Cre-negative/AdvDTA+ littermates (single cell RNAseq data indicates that all five Cre lines are expressed outside of the parvalbumin-positive PSNF3 proprioceptive DRG neurons) ( Figure 3A). The Randall-Selitto assay on the tail showed that noxious mechanosensation was significantly reduced in the Tmem45b Cre+/AdvDTA mice ( Figure 3B). Tmem45b is expressed in a subset of Na _v_1.8 positive neurons, specifically the six non-peptidergic neuronal subsets (PSNP1-6) ( Table 1). Punctate mechanical sensitivity, as assessed using von Frey filaments, was not different to controls for the Tmem45b, Th, Ntrk2 and Ntng1 Cre+/AdvDTA mice although surprisingly, increased sensitivity was observed in the CGRP Cre+/AdvDTA mice ( Figure 3C). Noxious thermal pain thresholds were assessed by the Hargreaves’ and hot plate tests and for both assays, CGRP Cre+/AdvDTA mice showed significant hyposensitivity to noxious heat ( Figures 3D and 3E). However, CGRP Cre+/AdvDTA mice did not show significant higher cold pain thresholds in the cold plantar assay, although a trend was observed ( Figure 3F). The AdvDTA reporter line was also tested in inflammatory and neuropathic pain assays. In the formalin test, no significant differences were observed for all AdvDTA+/Cre+ lines in the first phase ( Figure 4A). However, in the second phase, the AdvDTA+/CGRP Cre+ mice displayed significantly reduced nocifensive responses ( Figure 4A).

Next, we tested the AdvDTA+/Ntrk2Cre+ mice in the partial sciatic nerve ligation model to investigate the contribution of the PSNF1 and PSNF2 neuronal populations in the development of mechanical allodynia under neuropathic pain conditions. As expected mechanical hypersensitivity developed in the control littermates at 7 days and 16 days post-surgery ( Figure 4B). However, the AdvDTA+/Ntrk2Cre+ mice did not develop mechanical hypersensitivity, highlighting the importance of the PSNF1 and PSNF2 neuronal subsets to mechanical allodynia in the partial sciatic nerve ligation (PSL) neuropathic pain model.

## Discussion

Genetic tools have made a dramatic contribution to a better understanding of the nervous system. Thus human genetic studies have identified causative mutations for a variety of disorders ^29^, whilst gene and cell silencing ^30^ and in particular activity-based reporter systems such as CANE have proved extraordinarily informative ^31^.

The complexity of sensory neurons that play a key role in pain pathways has been highlighted by single cell transcriptional analysis ^3^. This suggests that there are at least 18 subsets for sensory neurons in mice. Importantly very similar subsets of sensory neurons are present in primates ^7^, reinforcing the view that mouse genetic studies are likely to be relevant and useful for understanding human peripheral pain pathways ^32^. 

Early experiments using a Na _v_1.8 Cre-recombinase to release diphtheria toxin and delete many peripherin-positive sensory neurons produced significant modality specific loss of function ^28^. Recent GCaMP studies are also consistent with the presence of a high proportion of modality-specific sensory neurons although there remains considerable debate on this topic ^6^.

Linking transcriptional changes in pain states to particular sets of sensory neurons has proved problematic ^33^. There is clearly extensive redundancy in pain mechanisms for example in temperature sensing ^34^ and cold sensation ^35^, whilst inflammation or damage changes the expression patterns of many transcripts including those encoding neuropeptides that are known to have a role in pain pathways. In addition, cells of the skin play a role as primary sensors for many painful stimuli indirectly signalling tissue damage to sensory neurons ^36^. Neuro-immune interactions also play a major role in changing the gain of the pain system, and regulating nociceptive activity ^37^.

Despite these complexities, sets of sensory neurons with particular sensory modalities clearly exist, and we have assembled tools to address their specialised functional role. A major strength of this study is that we have exploited known and novel Cre lines that encompass all sensory neuron subsets, and generated an advillin floxed stop diphtheria toxin mouse that will release the toxin only in sets of sensory neurons in the presence of Cre recombinase. A limitation of the study is that there is random integration of the BAC into the genome of the reporter mice with the potential for leaky expression. An improvement would be to generate a knockin mouse line under the advillin promoter with expression of diphtheria toxin under the control of a Cre-dependent FLEx switch.

Expression patterns were analysed with tomato reporter mice and preliminary phenotyping has been carried out analysing acute pain in mice in which sets of sensory neurons have been deleted. This background information should prove useful as a platform for more complete analyses of pathways involved in pain states that are clearly mechanistically different, for example cold allodynia and chronic inflammatory pain ^38^. The role of neurons normally associated with proprioception and innocuous sensation can also be addressed. Recent studies have demonstrated a significant efferent role for sensory neurons in immune responses, and conditions such as psoriasis ^39^. Dissecting out the new roles of sets of peripheral sensory neurons in regulating immune responses can thus also be addressed with the assembled mouse lines.

## Data availability

### Underlying data

Open Science Framework: Tools for analysis and conditional deletion of subsets of sensory neurons. https://doi.org/10.17605/OSF.IO/KTHDU ^9^.

This project contains the following underlying data:

- Raw values for behavioural experiments (including gender-specific values) underlying Figure 2– Figure 4, in open access CSV format
- Raw unedited microscopy images for Figure 1
- Supplementary Methods.pdf (contains raw pictures of gels from PCR testing)
- Figure 2C – qPCR CT values.csv (raw Ct values for all samples and replicates from RT-PCR)

### Extended data

Open Science Framework: Tools for analysis and conditional deletion of subsets of sensory neurons. https://doi.org/10.17605/OSF.IO/KTHDU ^9^. 

This project contains the following extended data:

- Supplementary Figure 1.pdf
- Supplementary Figure 2 (from v6).pdf
- Supplementary Figure Legends.pdf
- Supplementary methods.pdf

### Reporting guidelines

Open Science Framework: *ARRIVE checklist for ‘Tools for analysis and conditional deletion of subsets of sensory neurons’.* https://doi.org/10.17605/OSF.IO/KTHDU ^9^.

Data are available under the terms of the Creative Commons Zero "No rights reserved" data waiver (CC0 1.0 Public domain dedication).
