# Supplementary material for: Tools for analysis and conditional deletion of subsets of sensory neurons
Source: Wellcome Open Res. 2021 Sep 30;6:250. [Version 1] doi: 10.12688/wellcomeopenres.17090.1 (PMC8817070; doi:10.12688/wellcomeopenres.17090.1)
Supplement: Peer review history

# Peer review: Reviewer response for version 1

**DOI:** 10.21956/wellcomeopenres.18877.r47924

**Published:** 2022-02-17

**Authors:** Felix Viana

**Affiliations:** 1 Instituto de Neurociencias de Alicante, Universidad Miguel Hernandez-CSIC, San Juan de Alicante, Spain; 2 Consejo Superior de Investigaciones Científicas (CSIC), Madrid, Spain

The authors introduce a novel transgenic mouse line that allows the ablation of subpopulations of primary sensory neurons by selective expression of diptheria toxin. The advantage, compared to previous methods, is the fact that, by using the Advillin promoter, they can restrict the ablation to primary sensory neurons, avoiding possible confounding effects of eliminating other cell types also expressing the chosen Cre-driver. For example, it was recently shown that CGRP is also expressed in a subset of dorsal horn interneurons, and their selective activation or ablation has behavioral consequences (Löken *et al.*, DOI: 10.7554/eLife.59751). ^1^ The authors could cite these and other relevant literature to strengthen the point for developing this new tool. With the tool in hand, the authors explored the behavioral effects of ablating different subsets of primary sensory neurons and noted some differences. I am sure these approaches will be extended in future studies.

 As a way of background, the authors briefly rant against polymodality, in the introduction and the discussion, but, I am not convinced that ablation studies can help much in establishing whether a particular neuron or a particular subpopulation is polymodal or not. The lack of a phenotype for a given submodality (e.g. mechanosensitivity) after eliminating, even a large subset of neurons, can be explained by redundancy or overlapping expression of sensors. Other approaches are needed to settle this question. A brief review on this topic, written with my colleague, Prof. Carlos Belmonte, some years ago can be found in DOI: 10.1186/1744-8069-4-14. ^2^ Depending on the lens you use, the view can be clear or blurry. Rather than simply showing that the expression of the reporter and the driver/target marker gene overlap (Figure 1) reasonably well (some additional statistics may convince the more skeptic reader), I wished the authors went directly after the important goodies: how effective is the approach in ablating the different subpopulations. To be fair, they tested it with the Nav1.8 Cre line (figure 2) and the results are quite impressive. I guess that demonstrating selectivity and lack of effect on target populations would also be an important aspect to establish in the future.

 In my opinion, the authors should provide further details in the description of their behavioral methods. Some of their descriptions are too sparse and would not allow the proper replication of the experimental conditions. For example, when describing their noxious cold test, they simply refer to a published article (Brenner *et al.*, 2012, DOI: 10.1371/journal.pone.0039765). ^3^ However, in that study, the authors used three different glass thicknesses, which has a notable impact on the cooling rate of the surface and the withdrawal latency. Are their results comparable? Difficult to judge without knowing the thickness of the glass.

 Another aspect of the behavioral analysis used I find a bit trickier. They state in the methods that “control and test groups were assayed on the same day”. Presumably, this is important to minimize uncontrolled sources of variability in pain assessment in non-verbal species (DOI: 10.30802/AALAS-CM-19-000042). ^4^ However, in the analysis performed on figure 4 this is no longer considered and the group of control animals includes, apparently, all the controls (this group is much larger than the rest!), and the mean and SD obtained is the average for all the groups. Would the results/conclusion obtained be different if each of the groups was evaluated against the corresponding control? I don’t know but it would be relevant information. An expert in statistics, which I am not, can probably provide some advice.

Is the work clearly and accurately presented and does it cite the current literature?

Partly

If applicable, is the statistical analysis and its interpretation appropriate?

I cannot comment. A qualified statistician is required.

Are all the source data underlying the results available to ensure full reproducibility?

Yes

Is the study design appropriate and is the work technically sound?

Yes

Are the conclusions drawn adequately supported by the results?

Yes

Are sufficient details of methods and analysis provided to allow replication by others?

Partly

Reviewer Expertise:

Sensory transduction, pain neurobiology, role of ion channels in neuronal excitability

I confirm that I have read this submission and believe that I have an appropriate level of expertise to confirm that it is of an acceptable scientific standard.

## References

[1] : Contribution of dorsal horn CGRP-expressing interneurons to mechanical sensitivity. *eLife* .2021;10: 10.7554/eLife.59751 10.7554/eLife.59751 PMC824513034061020

[2] : Molecular and cellular limits to somatosensory specificity. *Mol Pain* .2008;4: 10.1186/1744-8069-4-14 14 10.1186/1744-8069-4-14 18419827PMC2386863

[3] : A novel behavioral assay for measuring cold sensation in mice. *PLoS One* .2012;7(6) : 10.1371/journal.pone.0039765 e39765 10.1371/journal.pone.0039765 22745825PMC3382130

[4] : A Review of Pain Assessment Methods in Laboratory Rodents. *Comparative Medicine* .2019;69(6) : 10.30802/AALAS-CM-19-000042 451-467 10.30802/AALAS-CM-19-000042 PMC693569831896391

# Peer review: Reviewer response for version 1

**DOI:** 10.21956/wellcomeopenres.18877.r47925

**Published:** 2022-02-08

**Authors:** Cruz Miguel Cendán Martínez

**Affiliations:** 1 Department of Pharmacology, Faculty of Medicine, Biomedical Research Center, Institute of Neuroscience, Parque Tecnológico de Ciencias de la Salud, University of Granada, Granada, Spain

Santana-Varela and colleagues have established new tools to study the specialised functional role of sets of sensory neurons. The authors have performed a series of elegant experiments to delete sensory neuron-specific Advillin which is expressed in all sensory neurons. The Advillin DRG promoter has been crossed with several new inducible Cre recombinase lines and behaviourally studied. The types of pain models used are wide and appropriate and explore some of the most common acute pain modalities such as mechanical and thermal pain. Also, the new Cre lines has been tested in the formalin test (inflammatory pain) and one of these lines (Ntrk2) was evaluated for neuropathic pain. However, as the authors mentioned in the manuscript, a more complete analyses of phenotyping of these mice including cold allodynia or chronic inflammatory pain would be necessary to characterize these lines. Nevertheless, these genetic tools will be useful and important for understanding the peripheral pain pathways.

**Comments for the Authors:**

- It is not clear if the same animal was tested once in only a pain model or in several of them. If the latter, it would be relevant to describe how much time passed between behavioural measuring.
- Since the legends in figure 3 are the same for all graphs and are very difficult to see because they are so small, perhaps putting a larger general legend above the figure would make it easier to read.

Is the work clearly and accurately presented and does it cite the current literature?

Yes

If applicable, is the statistical analysis and its interpretation appropriate?

Yes

Are all the source data underlying the results available to ensure full reproducibility?

Yes

Is the study design appropriate and is the work technically sound?

Yes

Are the conclusions drawn adequately supported by the results?

Yes

Are sufficient details of methods and analysis provided to allow replication by others?

Yes

Reviewer Expertise:

Behavioural pain research

I confirm that I have read this submission and believe that I have an appropriate level of expertise to confirm that it is of an acceptable scientific standard.

# Peer review: Reviewer response for version 1

**DOI:** 10.21956/wellcomeopenres.18877.r47926

**Published:** 2022-02-04

**Authors:** Uhtaek Oh

**Affiliations:** 1 Korea Institute of Science and Technology, Seoul, South Korea

Dorsal-root ganglion (DRG) neurons are very heterogeneous in size as well as in physiological functions including touch sensation, proprioception, pain, and itch. To dissect the roles of subsets of DRG neurons, the authors developed mouse lines that lacked specific subsets of DRG neurons. They developed Cre recombinase mouse lines in key marker genes that define subsets of DRG neurons. These Cre mice were crossed with transgenic mice that overexpressed DTA in DRG neurons. The authors confirmed the specific targeting of DTA to subtypes of DRG neurons. Then, the authors applied several pain tests to these mice lines, such as CGRP(Cre+/AdvDTA), Th(Cre+/AdvDTA), Tmem45b(Cre+/AdvDTA), Ntng1(Cre+/AdvDTA), and Ntrk2(Cre+/AdvDTA) to characterize the pain modality of the subsets of DRG neurons. In this study, a specific group of DRG neurons was also nicely ablated. This study contributes to dissecting the diverse roles of subsets of DRG neurons. The molecular and genetic techniques are good to test the hypothesis. Experiment results are significant. There are some points that I would like to comment on:

1. It would be nice if the authors discuss and compare these results with previous reports. For example, CGRP was known to play an important role in noxious heat sensitivity or inflammatory pain ^1^^,^^2^. TrkB-positive neurons were also ablated to investigate its role in mechanical pain ^3^.
2. The labels and numbers in the figures are too small to read. It would be better if the labels are enlarged.

Is the work clearly and accurately presented and does it cite the current literature?

Yes

If applicable, is the statistical analysis and its interpretation appropriate?

Yes

Are all the source data underlying the results available to ensure full reproducibility?

Yes

Is the study design appropriate and is the work technically sound?

Yes

Are the conclusions drawn adequately supported by the results?

Yes

Are sufficient details of methods and analysis provided to allow replication by others?

Yes

Reviewer Expertise:

Pain signalling mechanisms, Anoctamins, Tentonins

I confirm that I have read this submission and believe that I have an appropriate level of expertise to confirm that it is of an acceptable scientific standard.

## References

[1] : Variable sensitivity to noxious heat is mediated by differential expression of the CGRP gene. *Proc Natl Acad Sci U S A* .2005;102(36) : 10.1073/pnas.0503264102 12938-43 10.1073/pnas.0503264102 16118273PMC1200271

[2] : Inhibiting endocytosis in CGRP+ nociceptors attenuates inflammatory pain-like behavior. *Nature Communications* .2021;12(1) : 10.1038/s41467-021-26100-6 10.1038/s41467-021-26100-6 PMC849041834608164

[3] : Control of mechanical pain hypersensitivity in mice through ligand-targeted photoablation of TrkB-positive sensory neurons. *Nature Communications* .2018;9(1) : 10.1038/s41467-018-04049-3 10.1038/s41467-018-04049-3 PMC591560129691410

# Peer review: Reviewer response for version 1

**DOI:** 10.21956/wellcomeopenres.18877.r46341

**Published:** 2021-11-01

**Authors:** Mohammed A. Nassar

**Affiliations:** 1 Department of Biomedical Science, University of Sheffield, Sheffield, UK

The authors developed several new inducible Cre lines for targeting specific subsets of sensory neurons *in vivo*. The choice of driver promotors is based on genes that were found to be markers of specific subsets of sensory neurons by single cell sequencing of DRG neurons. The generated Cre lines were all inducible allowing genetic manipulations in adult mice. Inducible Cre expressed was demonstrated by a dTomato reporter line. The pattern of Cre activity was then compared to the mRNA expression of the driver genes. To demonstrate the applicability of the Cre lines for pain research, the authors used them to ablate the targeted neuronal populations. They generated a new DTA mouse line that is restricted to DRG neurons to reduce the possibility of cell death in any other tissue. Double transgenic mice were subjected to pain testing.

 Undoubtedly, the generated Cre lines are important for expanding our understanding of the role of the various subtypes of sensory neurons in plain conditions. The authors used an inducible Cre which is very appropriate to avoid the well-known disadvantages of genetic manipulations through development. It was great to note that the behavioural testing was carried out by researchers blind to the mice’s genotype.

**Comments for the Authors:**

1. The panels in figure 2 are too small, I had to enlarge the PDF to 400% to be able to clearly see the labelling. Would be it be possible to enlarge the panels?
2. In the first results paragraph, the authors state that there is a “large overlap” between the mRNA expression of the target and dTomato expression. It would be better if this was quantified as the percent of dtomot+/target+. The panels indicate that Cre may not be active in all the target neurons.
3. It is not clear why only the Ntrk2 line was tested for neuropathic pain development.

Is the work clearly and accurately presented and does it cite the current literature?

Yes

If applicable, is the statistical analysis and its interpretation appropriate?

Yes

Are all the source data underlying the results available to ensure full reproducibility?

Yes

Is the study design appropriate and is the work technically sound?

Yes

Are the conclusions drawn adequately supported by the results?

Yes

Are sufficient details of methods and analysis provided to allow replication by others?

Yes

Reviewer Expertise:

Using transgenic mice to study pain

I confirm that I have read this submission and believe that I have an appropriate level of expertise to confirm that it is of an acceptable scientific standard.
